# Image-guided MALDI mass spectrometry for high-throughput single-organelle characterization

**DOI:** 10.1038/s41592-021-01277-2

**Published:** 2021-09-30

**Authors:** Daniel C. Castro, Yuxuan Richard Xie, Stanislav S. Rubakhin, Elena V. Romanova, Jonathan V. Sweedler

**Affiliations:** 1 Department of Molecular and Integrative Physiology, University of Illinois at Urbana-Champaign, Urbana, IL USA; 2 Beckman Institute for Advanced Science and Technology, University of Illinois at Urbana-Champaign, Urbana, IL USA; 3 Department of Bioengineering, University of Illinois at Urbana-Champaign, Urbana, IL USA; 4 Department of Chemistry, University of Illinois at Urbana-Champaign, Urbana, IL USA; 5 Neuroscience Program, University of Illinois at Urbana-Champaign, Urbana, IL USA

**Keywords:** Endocrinology, Mass spectrometry, Metabolomics, Metabolomics, Cellular neuroscience

## Abstract

Peptidergic dense-core vesicles are involved in packaging and releasing neuropeptides and peptide hormones—critical processes underlying brain, endocrine and exocrine function. Yet, the heterogeneity within these organelles, even for morphologically defined vesicle types, is not well characterized because of their small volumes. We present image-guided, high-throughput mass spectrometry-based protocols to chemically profile large populations of both dense-core vesicles and lucent vesicles for their lipid and peptide contents, allowing observation of the chemical heterogeneity within and between these two vesicle populations. The proteolytic processing products of four prohormones are observed within the dense-core vesicles, and the mass spectral features corresponding to the specific peptide products suggest three distinct dense-core vesicle populations. Notable differences in the lipid mass range are observed between the dense-core and lucent vesicles. These single-organelle mass spectrometry approaches are adaptable to characterize a range of subcellular structures.

## Abstract (web summary)

This article presents a workflow for image-guided MALDI MS characterization of peptide and lipid content in single organelles.

## Main

Organelles are one of the smallest structural units that influence the functional, morphological and biochemical characteristics of different cell types. Chemical analysis of individual organelles is challenging due to their attoliter volumes, the wide dynamic range of analyte concentrations and the need for sophisticated isolation procedures, thereby limiting our understanding of their chemical heterogeneity. Mass spectrometry (MS) imaging has begun being used for both cellular and some subcellular analyses in discovery-based studies^1–3^ but is limited in throughput and spatial resolution for organelle measurements. Alternatively, both cells^4–6^ and organelles^7^ of interest can be isolated and placed on a glass slide for subsequent MS measurement. Though we used this approach to assay the peptides within individual organelles^7^, it was limited in throughput and difficult to automate due to the manual positioning of each individual organelle before measurement.

**Fig. 1:**
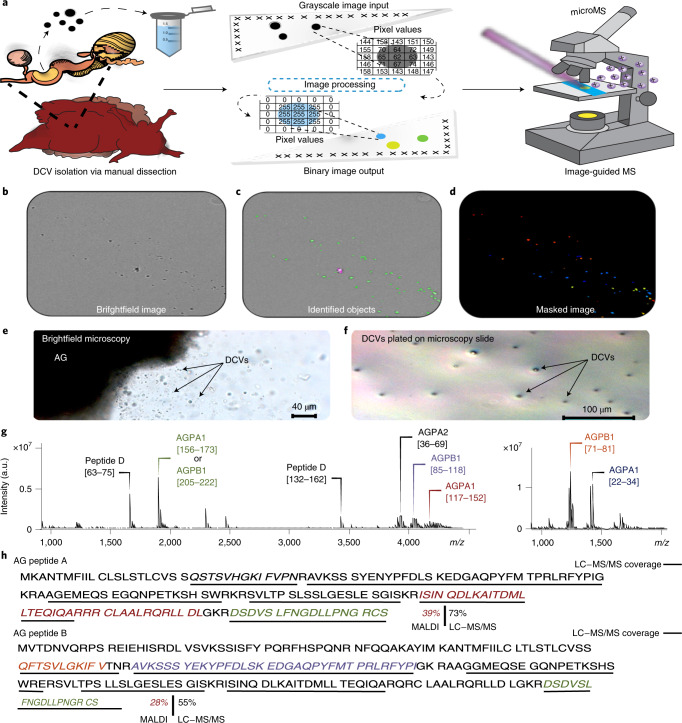
High-throughput workflow for label-free single-DCV targeting and MS analysis. **a**, Schematic of MALDI MS workflow for high-throughput single-DCV measurements. **b**, Brightfield image of DCVs distributed on a glass slide. **c**, Identification of primary objects (DCVs) using image-processing software. **d**, Masked image output of identified DCVs. The colored spots represent primary objects (DCVs) recognized as ‘foreground’ and are marked with a maximal pixel intensity value. Anything not identified as an object is treated as ‘background’ and is set to a zero-pixel intensity value. **e**, Brightfield image of mechanically induced DCV release from the AG. **f**, DCVs plated on a glass slide for relative DCV density estimation using brightfield microscopy. Each slide held DCVs from three animals (biological replicates) and a total of three slides (technical replicates) were prepared, where 598 DCVs were measured. **g**, Mass spectra demonstrating the coverage of AG peptides detected in single-DCV measurements. **h**, AGPA1 (XP_012945143.1) and AGPB1 (XP_012945142.1) prohormone sequences with corresponding MALDI MS-detected peptides italicized and font colored to match the annotated spectra in **g**. AG peptide assignments were validated using LC–MS/MS and performed on AG extracts (*n* = 3). The peptides detected by LC–MS/MS are underlined in black. a.u., arbitrary units.

A recent enhancement to single-cell measurement^8^ involves scattering the cells of interest onto a microscope slide, determining their locations via fluorescence microscopy and then targeting selected locations with matrix-assisted laser desorption/ionization (MALDI) MS, allowing tens of thousands of cells to be assayed. Here we adapted the single-cell approach to single organelles, which required three enhancements: (1) improved object targeting approaches, (2) optimized analyte detection using high-resolution MS and (3) unsupervised data analysis workflows to characterize organelle heterogeneity. These advances allowed us to use MALDI Fourier-transform ion cyclotron resonance MS for the high-throughput simultaneous detection of both peptide and lipid species in 0.5- to 2-μm-diameter dense-core vesicles (DCVs) and electron lucent vesicles (LVs) isolated from the exocrine atrial gland (AG) (Fig. 1a–f) and red hemiduct of *Aplysia californica*, respectively. By developing single-organelle sampling techniques that can be conducted in a high-throughput manner, we observed subtypes of DCVs defined by their overlapping but distinct peptide content. We also identified a peptide prohormone not previously known to localize within DCVs^9–11^, as well as large differences in the contents between the DCVs and LVs. Our method revealed post-translational proteolytic processing of AGPB1 (XP_012945142.1), AGPA1 (XP_012945143.1) and AGPA2 (XP_012945134.1) prohormones, with peptide structures validated by liquid chromatography–tandem mass spectrometry (LC–MS/MS). Additionally, a novel prohormone, AG Peptide D, was characterized (Supplementary Figs. 1 and 2 and Supplementary Table 1). Importantly, our workflow is extendable to multiple imaging modalities such as scanning electron microscopy (Supplementary Fig. 3) and instrumentation that uses a piezo linear stage and camera, providing an avenue for the analysis of targets that are smaller than the wavelength of light.

## Results

### Image-guided MALDI MS approach

To adapt single-cell approaches to objects that are tenfold smaller in diameter than cells measured previously, we created a method to enhance identification of micrometer-sized object locations while improving analyte detectability by minimizing the number of interfering chemicals that could reduce the ionization efficiency of our analytes of interest. Traditionally, single-cell targeting requires chemical labeling to mark objects of interest, but introduction of exogenous chemicals during single-organelle sample preparation is problematic due to the 1,000-fold less material present. Paraformaldehyde fixation is a hallmark step in most staining protocols, but paraformaldehyde fixation crosslinks DNA and peptides/proteins, rendering them poorly ionizable and undetectable by MS. By measuring the pixel properties produced by DCVs under brightfield conditions, we compiled sequences of image algorithms (Supplementary Fig. 4) that selectively identify the pixels produced by DCVs versus the background (Fig. 1b–d), allowing identification of the spatial locations of individual DCVs without the need for chemical labeling. The axial resolution of light microscopy limits this approach to the analysis of vesicles that are approximately 500 nm in diameter or larger. However, nonoptical approaches such as scanning electron microscopy can be applied, providing a resolution down to 10 nm, allowing targeting of nanometer-sized objects (Supplementary Fig. 3). A three-step approach was developed for isolation of a representative number of vesicles without loss of chemical detail, which is critical for the high-throughput preparation of organelle samples. In this approach, a volatile, isosmotic-ammonium acetate buffer was deposited onto an indium tin oxide (ITO)-coated glass slide, into which a small aliquot of artificial sea water (ASW) solution containing the vesicles was deposited. The vesicles were allowed to sediment and adhere to the glass surface before the solution was aspirated, leaving a large number of visually intact vesicles distributed across the slide. After identifying the vesicles on the slide using the described pipeline, an image mask of the identified objects was created. The image mask marks the vesicles with a maximal pixel intensity and forces the remaining pixels to a zero-pixel intensity, creating a binary image output (Fig. 1d). With the vesicles marked by clusters of maximal pixel intensity values, the binary image created from the masked image output can be used for identification of the spatial locations of vesicles on the slide for subsequent analysis via MALDI MS. A 200-μm-distance filter was applied to remove objects from the target list that are closer than 200 μm to each other, ensuring the 100-μm-diameter MALDI laser spot size did not overlap with multiple vesicles (Supplementary Fig. 5).

### High-throughput DCV detection and characterization

**Fig. 2:**
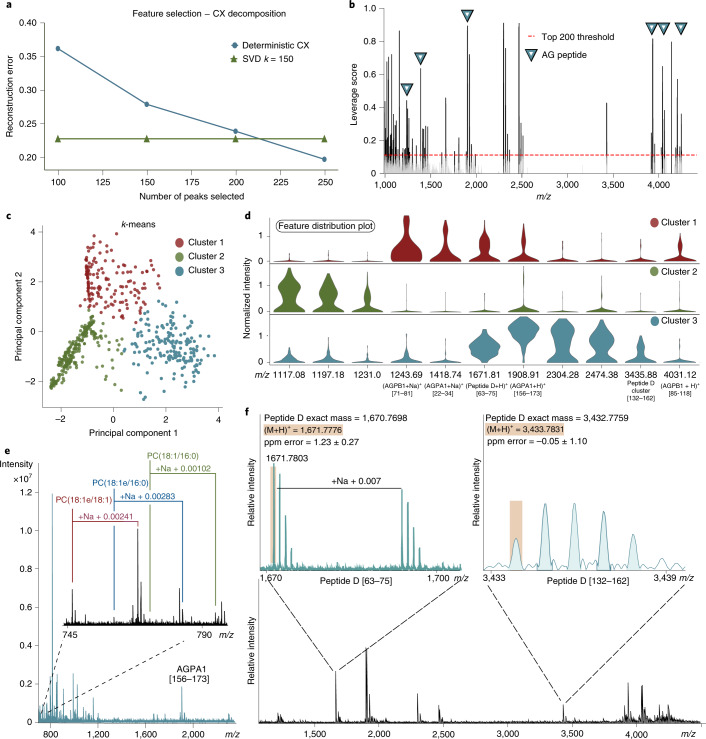
Chemical heterogeneity of DCV populations: unsupervised data analysis, simultaneous acquisition of analyte classes and detection of novel Peptide D. **a**, Deterministic CX was used to select 200 mass spectral features to improve data interpretation. CX decomposition was applied for feature selection where the best low-rank approximation was provided through singular value decomposition with a rank parameter *k* = 150, which was determined by the reconstruction errors with different rank parameters. Rank *k* = 150 has a reconstruction error less than 25% of the original dataset. **b**, ‘Statistical leverage scores’ for all spectral features were computed and plotted against the *m*/*z* axis. Multiple detected AG peptides can be seen with high leverage scores and are annotated with blue triangles. The ‘Statistical leverage score’ for each feature indicates its influence on the best low-rank fit of the data matrix. Selecting the top 200 features ensured that CX decomposition closely approximated the best low-rank fit of the original data matrix while removing uninformative information. **c**, Results of *k*-means clustering of the dataset containing 200 selected features with the highest statistical leverage score. **d**, Violin plots visualizing a subset of the selected features and their relative distribution in each cluster, with known AG peptides marked. **e**, Simultaneous detection of PC(18:1/16:0), PC(18:1e/16:0) and PC(18:1e/18:1) with their corresponding sodiated adducts annotated. **f**, The novel prohormone, Peptide D, was identified by MALDI MS mass-match assignment of Peptide D [63–75] and Peptide D [132–162]. SVD, singular value decomposition.

A total of 598 DCVs were analyzed and, after cross-refencing the single-DCV spectra with the LC–MS/MS data, over 50 mature full-length peptides were assigned by peptide mass fingerprinting to eight known prohormones and one novel prohormone (Supplementary Table 2). As a result, the peptides AGPA2 [36–69], AGPB1 [85–118], AGPA1 [117–152], AGPA1 [156–173] (or identical peptide AGPB1 [205–222]), N-terminal peptide AGPA1 [22–34] and the truncated N-terminal peptide AGPB1 [71–81] lacking the C-terminal Thr-Asn^7^ were detected, resulting in 39% and 28% coverage of AGPA1 and AGPB1 prohormones (Fig. 1g,h), respectively. Lastly, by using an *m*/*z* range of 150–4,500, we were able to perform simultaneous detection of both peptide and lipid species in individual DCVs. Three phosphatidylcholine (PC) lipid species detected by mass-match assignment include: PC(18:1/16:0), PC(18:1e/16:0) and PC(18:1e/18:1), with respective assignment mass errors of −1.62 ± 0.65 ppm, −2.59 ± 0.06 ppm and −1.55 ± 0.21 ppm (Fig. 2e). The three detected lipids were previously identified in *A. californica* neurons using MALDI MS^12^.

Differential packaging of peptides from the same prohormone into different vesicles was shown in the bag cell neurons of *A. californica* using immunogold staining approaches^13^, although this has not been demonstrated in other *A. californica* cell types. Here, we considered whether the different peptide complements may be from differential proteolytic processing of AGPB1, AGPA1 and AGPA2 prohormones. Therefore, CX decomposition, an unsupervised statistical approach, was applied to select statistically important features while removing redundant and uninformative ones for improved downstream data interpretation (Supplementary Figs. 6 and 7). CX decomposition based on statistical leverage scores was used to select the top 200 features^14^ (Fig. 2a,b and Supplementary Fig. 8). Next, using the selected features, the DCVs were partitioned into three groups using the *k*-means clustering algorithm, for which the number of groups was determined by calculating the Within Cluster Sum of Squares (Fig. 2c). Significantly different features were obtained by performing a Wilcoxon rank-sum test for each individual cluster against the others (Supplementary Fig. 9). The violin plots (Fig. 2d) highlight selected mass spectral features and their distribution in the different DCV cluster types where known AG peptides are labeled. The N-terminal peptides, AGPB1 [71–81] and AGPA1 [22–34], and the novel Peptide D [63–75] (Fig. 2f), are shown to be defining features in Cluster 1 when compared with Cluster 2, whereas Cluster 3 demonstrates localization of multiple expected AG peptides from the prohormone C termini, including AGPA1 [156–173], Peptide D [63–75], AGPB1 [85–118] and a peptide cluster belonging to Peptide D [132–162] (Supplementary Fig. 10). The data indicate that the N- and C-terminal peptides from these two prohormones are differentially packaged into a subset of DCVs, whereas the bioactive peptides that initiate the egg-laying process^15^, AGPA2 [36–69] and AGPB1 [85–118], are predominantly packaged into a separate subset of vesicles. An early pioneering study that quantified the amounts of AGPA1 [22–34], AGPA2 [36–69] and AGPB1 [85–118] in isolated intact granules from AG homogenates also suggested the possibility of packaging of AGPA1 [22–34] into a distinct class of secretory vesicle^15^. Perhaps this differential packaging of N-terminal products in the AG would be akin to what is also observed in the bag cell neurons where N- and C-terminal peptides are differentially packaged into two discrete vesicle classes that are targeted to different cellular locations^13^.

### Mass spectrometric characterization of LVs

**Fig. 3:**
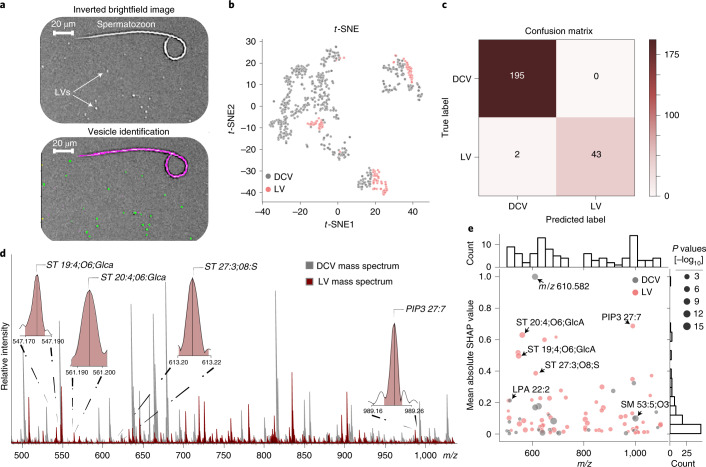
Comparison between two distinct vesicle types, LVs and DCVs. **a**, Top, digitally inverted brightfield image showing LVs distributed across the glass slide. **a**, Bottom, modification of the image-processing pipeline allows multiple morphologically distinct objects to be identified and added or removed for subsequent analysis. Green areas represent objects accepted for analysis. Magenta outline represents an object (here a spermatozoon) removed from analysis. A small number of spermatozoa (annotated) typically collected during red hemiduct LV sample preparation were used to demonstrate the effectiveness of our object filtering approach. A total of 123 LVs were measured from the same three biological replicates used in the DCV isolation. **b**, *t*-SNE was performed on the initial dataset for visualization of LVs and DCVs using all mass spectral features. **c**, Confusion matrix of the prediction on the test data using a threefold validation. **d**, Representative LV mass spectrum (red) overlaid on a representative DCV mass spectrum (gray). Representative spectra were not preprocessed. A subset of mass spectral features determined important via SHAP are annotated in the LV mass spectrum. **e**, Mass spectral feature contribution plot showing the importance of features to the vesicle classification task across *m*/*z* 500–1,100 on the *x* axis. The *y* axis shows the mean absolute SHAP value for the corresponding *m*/*z* feature (normalized between 0 and 1). Gray dots represent features with elevated mean signal intensities in DCVs and pink dots represent features with elevated mean signal intensities in LVs. The different sized dots represent the respective *P* values (two-sided Wilcoxon rank-sum test) for each corresponding feature.

To demonstrate the suitability of our method to assay a variety of organelles, we extended it to another morphologically distinct vesicle type, LVs, from the adjacent red hemiduct of *A. californica*. Simple modification to the image-processing pipeline allows direct targeting of the LVs based on their different morphological characteristics (Fig. 3a). The LVs of the red hemiduct have a similar diameter of 0.5 to 2 µm but vary dramatically in ultrastructure; the LVs contain a double membrane structure, with the inner membrane forming cristae-like structures that are analogous to those found within the mitochondrion^9,16^. In contrast, DCVs from the AG contain a single membrane and exhibit a solid dense-core compared with the more relatively fluid interior of the LVs from the red hemiduct^9,16^. Due to the nonpeptidergic content of the LVs and the clear differences in ultrastructure between the LVs and DCVs, a machine learning model was trained for the differentiation between LVs and DCVs, focusing on the *m*/*z* range of 500–1,100. *t*-Distributed stochastic neighborhood embedding (*t*-SNE) was initially applied using the mass spectral features to visualize the data in a low-dimensional space (Fig. 3b). Next, gradient boosting trees were trained with a threefold validation, obtaining an accuracy of 98.6 ± 0.78% for the classification between LVs and DCVs (Fig. 3c). The most important features for the classification between LVs and DCVs (Fig. 3d) were selected via Shapley additive explanations (SHAP) through a previously described method^6^, where a total of 97 features with nonzero mean SHAP values from the model output were selected, with 36 features putatively identified by mass-match assignment (Fig. 3e). The model provided an improved classification performance through retraining on the selected features (Supplementary Fig. 11), displaying the differential lipid profiles between the two vesicle classes (Supplementary Figs. 12, 13 and 14). The data show the strong presence of multiple sterol lipid species to be of importance to their classification. The absence of sterol lipid species correlates with uniformly curved membranes^17,18^. Whereas when present in sufficient concentration, sterol lipid species will distribute asymmetrically in the lipid bilayer causing large, flat membrane regions that are separated by sharp curves in the membrane, creating negative membrane curvature. Interestingly, negative membrane curvature would promote the formation of the cristae-like invaginations that can be observed in the LVs^16,18^.

## Discussion

As the spatial resolution of MS advances from the single-cell level to include the single-organelle domain, new analytical approaches are being developed to unravel the complex microenvironments housed within single cells^19–21^. Capillary micro-sampling techniques coupled to electrospray ionization (ESI)–MS have been developed for the identification of peptides in individual cells and in some subcellular compartments of cells^11,22^. Though capillary micro-sampling ESI–MS techniques can identify peptides in subcellular compartments^23,24^, they require fine-tuning of the capillary position and controlling the volume extracted. These requirements are critical to single-organelle extraction and pose challenges to repeated sampling and throughput due to potential contamination from the cellular membrane, cytoplasm and other subcellular organelles^22^. Alternatively, our image-guided MALDI MS approach provides direct single-organelle targeting using point-based image registration.

In conventional MS imaging, as spatial resolution increases, so does acquisition time. Therefore, to maintain the throughput required for characterizing large populations of individual organelles, we are not imaging slides containing the vesicles. Instead, the organelles themselves are isolated and selectively targeted using the MALDI laser. Our high-throughput, label-free, single-organelle approach enables simultaneous characterization of both the peptide and lipid contents of hundreds of intact 0.5- to 2-µm-diameter DCVs and LVs. Because our approach uses a combination of freeware and instrumentation that is commonly available to researchers, it is adaptable to many experimental designs, including other organelle types such as microvesicles, which can range from 100 nm up to 1 μm in diameter^25^.

## Methods

### General

All chemicals were obtained from Sigma-Aldrich unless specified otherwise.

### Vesicle isolation

*A. californica* (150–200 g) were obtained from the National Resource for Aplysia and kept in a 14 °C aquarium filled with Instant Ocean (Aquarium Systems). Animal euthanasia was performed in accordance with the AVMA Guidelines for the Euthanasia of Animals: 2020 Edition (Section S6.3.1.1 Noninhaled agents for immersion). Animals were anesthetized using a 333 mM MgCl_2_ solution injected into the body cavity (50% volume/body).

For DCV isolation, the AG was isolated by manual dissection, and placed into a microcentrifuge tube containing 1 ml of ASW. Due to the holocrine release mechanism, DCV secretion from the AG was induced by gentle trituration with a polypropylene Pasteur pipette, releasing intact DCVs into the ASW solution. Next, 100 μl of 500 mM ammonium acetate solution was prespotted onto an ITO-unpolished float glass slide, Surface Resistivity = 70–100 Ω (Delta Technologies). Then, 50 μl of vesicle-containing ASW solution was pipetted into the 100 μl of ammonium acetate solution on the ITO-glass slide for a total volume of 150 μl. The resulting vesicle solution was then rinsed with 500 mM ammonium acetate with simultaneous aspiration of the solution, leaving DCVs seeded across the slide. All steps were visually monitored using an inverted microscope. Each slide held DCVs from three animals (biological replicates) and a total of three slides (technical replicates) were prepared. For LV isolation, the procedure used for the DCVs was repeated using red hemiduct tissue, with the other steps being the same as for the DCV isolation; 123 LVs were analyzed from the same three biological replicates used for the DCV isolation.

### Vesicle imaging

Brightfield images were acquired on an Axio Imager M2 (Zeiss) equipped with an AxioCam ICc 5, a ×0.63 camera adapter and a transmitted light visible light-light emitting diode lamp. Images were acquired in mosaic mode using a ×10 objective with 30% tile overlap. The resulting tiles were stitched before exporting in TIFF-file format using ZEN 2.0 Pro edition (Zeiss) software.

Scanning electron microscopy images were acquired using an FEI Quanta FEG 450 environmental scanning electron microscope (FEI). Images were acquired using an accelerating voltage of 10 kV, dwell time of 10 µs and working distance of 6.6 mm.

### Image-processing for vesicle recognition in microMS

We utilized biological image analysis software, including ImageJ^26^ and CellProfiler^27^, for vesicle pixel recognition on the brightfield microscopy image. Importantly, other image-processing software can be used for this process as well. CellProfiler pipeline modules were created to selectively mask the DCVs and LVs on the brightfield microscopy image to remove the background and retain just information on the vesicles of interest as the foreground. Masking the identified vesicles creates a binary image marking the pixel locations of vesicles on the glass slide. The microMS software was used to translate the pixel locations on microscopy images to physical coordinates of the mass spectrometer’s stage. A 200-µm-distance filter was then applied in microMS (removing vesicles located closer than 200 µm to each other from the target list). Alternatively, ImageJ was used as a simple thresholding strategy by measuring the difference between the vesicle (foreground) and background pixel intensity, which allows a threshold to be set, leaving only objects of interest for recognition in microMS.

### Matrix application

Matrix deposition for MALDI MS analysis was performed using a glass sublimation apparatus (Wilmad-LabGlass) filled with 2,5-dihydroxybenzoic acid as the MALDI matrix. The slide was attached to the cold-finger and vacuum was created using a rotary vane pump (Edwards Vacuum, model E2M30). The sublimation apparatus was placed on a sand bath preheated to 150 °C for 8 min. Matrix deposition was followed by recrystallization using 5% methanol. Recrystallization was performed using a 100 × 15-mm^2^ polystyrene Petri dish as a recrystallization chamber. The ITO-glass slide was attached to the top of the recrystallization chamber and a filter paper (Whatman Grade 1 Qualitative Filter Paper, Thermo Fisher Scientific) was wetted with 1 ml of 5% methanol. The chamber was sealed using tape and placed in an oven at 85 °C for 1.5 min. After removal of the recrystallization chamber from the oven, the slide was immediately removed from the recrystallization chamber and allowed to dry in a nitrogen chamber until analysis.

### MALDI MS measurements

High-throughput single-DCV and single-LV analyses were performed on a SolariX XR 7T Fourier-transform ion cyclotron resonance mass spectrometer equipped with an APOLLO II dual MALDI/ESI source (Bruker) using an *m*/*z* range of 150–4,500. Data were acquired at 1 M giving a mass resolution of 107,000 at *m*/*z* 535 and 19,070 at *m*/*z* 3,922, yielding a transient length of 0.721 s. The instrument was operating in positive-mode using a Smartbeam-II UV laser (Bruker) set to ‘Ultra mode’, which yields a 100-µm-diameter laser footprint. Each MALDI acquisition consisted of two accumulations comprised of 400 laser shots each, at a frequency of 1,000 Hz. DCV and LV stage coordinates and geometry files were generated using microMS as previously described^8^.

### Data preprocessing

Data preprocessing was performed using Compass Data Analysis 4.4.2 (Bruker) and MATLAB 2018b (MathWorks). Internal calibration using the exact mass of AG peptides was performed using AGPB1 [71–81] (*m*/*z* 1,221.6878), AGPA1 [22–34] (*m*/*z* 1,396.7225), AGPA1 [156–173] (*m*/*z* 1,908.8761), AGPA2 [33–69] (*m*/*z* 3,922.9478) and AGPB1 [85–118] (*m*/*z* 4,031.0053). Peak picking and peak export for statistical analysis in MATLAB 2018b were set to a signal-to-noise ratio of 5 with a relative intensity threshold of 0.01%. A nonuniform bin width was used for mass spectral alignment. For DCV data analysis, mass features were truncated at *m*/*z* 1,100 for downstream data analysis of only AG peptides. For LV and DCV vesicle classification, mass features in *m*/*z* 500–1,100 were selected for downstream data analysis. Internal calibration using AG peptides was not performed for the LV and DCV classification tasks. Using the target list provided by microMS, the pixel coordinates were used to find and crop the locations of individual vesicles across the original microscopy image. The corresponding mass spectra were matched with the appropriate vesicle for visual evaluation of corresponding single vesicles.

### CX decomposition and statistical analysis

Unsupervised approaches such as principal component analysis (PCA) and, more generally, matrix decomposition or factorization, are valuable data analysis tools to enable field-specific interpretation of high-dimensional datasets. However, the interpretation is limited due to the complicated eigenspace obtained from PCA, deterring our further understanding of the feature space of the data matrix. CX matrix decomposition is designed to obtain a low-rank approximation of the data in terms of actual rows or columns^14^. Given an *m* × *n* data matrix *A*, the algorithm decomposes it into an *m* × *c* matrix *C* and a *c* × *n* matrix *X*, where *C* is expressed by *c* number of column vectors of the original data. The statistical leverage scores are used to rank and select the columns of *C* from *A*, which can be obtained by

$$l_j = \mathop {\sum }\limits_{i = 1}^k v_{ji}^2$$

where *l*_*j*_ is the leverage score for the *j*th column/feature, *v*_*i*_ is the right singular vectors obtained by the singular value decomposition and *k* is the rank to be selected. *X* is then determined by minimizing the error:

$$\mathop {{\min }}\limits_X \left| {\left| {A - CX} \right|} \right|_F$$

The reconstruction error evaluation and the rank *k* selection are provided as Supplementary Fig. 8. Based on the evaluation, the top 200 features are selected to form the columns of the matrix *C*. *k*-means clustering, and the Wilcoxon rank-sum test, were performed using the Python-based open source package SCANPY^28^. The stacked violin plots, shown in Fig. 2d, of the normalized signal intensities represent the identified peak features across the three clusters, with the vesicle type (or cluster) assignments obtained by *k*-means clustering. The *y* axis is the root-mean-squared-normalized peak intensity and each violin contains the distribution of the normalized intensities of the corresponding features in the *x* axis.

### Peptide sequencing by LC–MS/MS

Peptide extracts (*n* = 3) were obtained by manually grinding entire AG tissue in 500 μl of acidified methanol followed by evaporation and reconstitution of each extract in 0.1% formic acid. A nanoElute (Bruker) ultra-high-pressure nano-flow chromatography system was coupled to a trapped ion mobility–quadrupole time-of-flight mass spectrometer (timsTOF Pro, Bruker) with a CaptiveSpray nano-electrospray ion source (Bruker) equipped with an external column oven. Mobile phases A and B were water with 0.1% formic acid (v/v) and acetonitrile with 0.1% formic acid (v/v), respectively. Samples were loaded onto a precolumn peptide trap (Acclaim PepMap 100 C18, 1 × 5 mm^2^, 5-µm particle size, Thermo Fisher Scientific) using solvent A for off-line desalting. Next, the trap was placed in-line with the analytical column and peptide separation was performed at 40 °C with a uniform flow of 300 nl min^−1^ on a C18 ReproSil AQ column (Bruker FIFTEEN, P/N no. 1842621: 150 mm × 75 µm, 1.9-µm particle size, pore size 120 Å) equilibrated at 2% B. A linear gradient of solvent B was applied as follows: 2–10% within 5 min, 10–50% in the next 115 min, followed by a washing step at 95% B and re-equilibration, during which data collection was not performed. The mass spectrometer was operated in parallel accumulation–serial fragmentation (PASEF) mode for peptide sequencing. The mass range for the precursor ion was set to *m*/*z* 100–1,700, ion mobility 1/*K*_0_ range 0.6–1.6 V s cm^−2^. Fragmentation was performed with 10 PASEF scans, cycle time of 1.1 s, during which collision energy varied linearly between 20 and 59 eV depending on precursor 1/*K*_0_ value within the set range. Active dynamic exclusion of precursor ions was set to 0.4 min.

### Peptide identification by bioinformatics

MS raw files were processed with PEAKS Online^29^ (Bioinformatics Solution) using the DeNovo, database (DB) and post-translational modification (PTM) protocols, sequentially. Peptide sequence tags obtained by the DeNovo process (80% average local confidence score cut-off) were searched against the *Aplysia* RefSeq database available from the NCBI (GCF_000002075.1). The protein database was filtered to include proteins up to 1,000 amino acids long. Search parameters for DB included: parent mass error tolerance 20.0 ppm, fragment mass error tolerance 0.03 Da, no enzyme, digest mode unspecific. Next, PTMs were identified by searching the data for amidation, acetylation (K) and acetylation N terminus, pyro-glutamylation from (Q) and (E), phosphorylation (STY) and half-disulfide bridge. The false discovery rate was determined by decoy fusion method and the threshold set to 1% for peptides. Proteins with −log_10_ *P* > 20 and at least one unique peptide are reported.

### Vesicle classification through machine learning

We adapted a previously described machine learning strategy^6^ for vesicle type classification to differentiate between DCVs and LVs based on their lipid contents. Features in the *m*/*z* range of 500–1,100 were used for the classification task. Gradient boosting trees were trained with a threefold validation; in each fold of the model performance, the metrics were computed to obtain classification accuracies, confusion matrices and receiver operating characteristic curves (Supplementary Fig. 11). The most contributing features to the classification task were selected via SHAP, a game theory approach for model explanations, and were obtained through a Python implementation of SHAP. A total of 97 features with nonzero mean SHAP values from the output of trained models were selected and 36 were putatively annotated by searching against the LIPID MAPS^30^ database with a 7-ppm tolerance. Models were then retrained with the SHAP-selected features as well as the annotated features to verify the discriminative ability of those features. *t*-SNE using the cosine distance was used to visualize the lipid differences between DCVs and LVs in a low-dimensional space.

### Reporting Summary

Further information on research design is available in the Nature Research Reporting Summary linked to this article.

## Online content

Any methods, additional references, Nature Research reporting summaries, source data, extended data, supplementary information, acknowledgements, peer review information; details of author contributions and competing interests; and statements of data and code availability are available at 10.1038/s41592-021-01277-2.

## Supplementary information

Supplementary Information Supplementary Figs. 1–14 and Tables 1 and 2.

Reporting Summary

## Data Availability

The data that support the findings of this study are publicly available via the Illinois Data Bank (10.13012/B2IDB-5949772_V1).

## Code availability

The code used in this study is available on GitHub (https://github.com/richardxie1119/DCV_DA).
