# Supplementary material for: Image-guided MALDI mass spectrometry for high-throughput single-organelle characterization
Source: Nat Methods. 2021 Sep 30;18(10):1233–8. doi: 10.1038/s41592-021-01277-2 (PMC8490150; doi:10.1038/s41592-021-01277-2)
Supplement: Supplementary file 1 — Supplementary Figs. 1–14 and Tables 1 and 2. [file 41592_2021_1277_MOESM1_ESM.pdf]

nature portfolio

https://doi.org/10.1038/s41592-021-01277-2

Supplementary information

Image-guided MALDI mass spectrometry for high-throughput single-organelle characterization

In the format provided by the authors and unedited

Supplementary Information

Image-guided MALDI mass spectrometry for high-throughput single-organelle characterization

Daniel C. Castro[1,4], Yuxuan Richard Xie[4,5], Stanislav S. Rubakhin[2,3,4], Elena V. Romanova[2,3,4], Jonathan V. Sweedler[1,2,3,4,5,*]

[1]Department of Molecular and Integrative Physiology, University of Illinois at Urbana-Champaign, Urbana, IL USA

[2]Department of Chemistry, University of Illinois at Urbana-Champaign, Urbana, IL USA

[3]Neuroscience Program, University of Illinois at Urbana-Champaign, Urbana, IL USA

[4]Beckman Institute for Advanced Science and Technology, University of Illinois at Urbana-Champaign, Urbana, IL USA

[5]Department of Bioengineering, University of Illinois at Urbana-Champaign, Urbana, IL USA

[*]Corresponding author, jsweedle@illinois.edu

Table of Contents

Supplementary Fig. 1: Identification and assignment of novel AG Peptide D prohormone S-2
Supplementary Fig. 2: Post-translational processing of novel AG Peptide D prohormone S-2
Supplementary Table 1: AG Peptide D prohormone-supporting peptides detected by LC-MS/MS S-3
Supplementary Fig. 3: DCV recognition using scanning electron microscopy S-4
Supplementary Fig. 4: CellProfiler parameters S-5
Supplementary Fig. 5: Representative mass spectra and brightfield DCV image S-5
Supplementary Fig. 6: t-SNE projection by analysis day S-6
Supplementary Fig. 7: Clustering without data dimensionality reduction S-7
Supplementary Fig. 8: Singular value decomposition of the processed dataset prior to feature selection S-7
Supplementary Fig. 9: Wilcoxon rank-sum test and feature value distributions S-8
Supplementary Fig. 10: CX decomposition-identified peptide cluster S-9
Supplementary Table 2: LC-MS/MS validation of peptides in AG DCVs by MALDI MS S-10
Supplementary Fig. 11: ROC curves obtained from 3-fold validation S-12
Supplementary Fig. 12: Volcano plot of the differential lipid features between DCVs and LVs S-12
Supplementary Fig. 13: DCV and LV t-SNE feature distributions S-13
Supplementary Fig. 14: Mass spectral profile comparisons between DCVs and LVs. S-14
References. S-14

```
pir||A28108         1 ------------------------------------------------------------   0
XP_012943319.1      1 MSGPAATLVFLLVSLLSLNSQLTQVAGARSHDAGIYRHATREDVLGTSDALESEMLGHPK  60

pir||A28108         1 ------------------------------------------------------------   0
XP_012943319.1     61 RECRFITCVFWDRIDRRNSGVTEDVISEDYEGPQGSVLRNLQEQKRAINTLRKLTEILKP 120

pir||A28108         1 -----------TQRLRLGGHQLTEELDKAAKQYAYLK-----------------------  26
XP_012943319.1    121 DQQQIVTTRKRTCRLRLGGHCLTEELDKAAKQYAYLKSGKSPGRRRRETGNAAILENHDR 180
                                 * ******* ****************

pir||A28108        27 --                                                            26
XP_012943319.1    181 YE                                                           182
```

Supplementary Fig. 1. Identification and assignment of novel AG Peptide D prohormone. Sequence alignment by Clustal Omega[1] indicates 92% residue identity among partial AG peptide D1 (pir||A28108) and predicted uncharacterized protein LOC101856757, XP_012943319.1; conserved residues are highlighted in green. In the process of the LC-MS/MS data analysis, a novel prohormone was assigned from previously unidentified and uncharacterized, predicted protein XP_012943319.1 by its sequence similarity to previously reported peptide of unknown origin, tentatively named Peptide D1 and determined to originate from the unknown precursor[2]. We now name this new prohormone AG Peptide D.

>XP_012943319.1 PREDICTED: uncharacterized protein LOC101856757 [Aplysia californica]

*MSGPAATLVFLLVSLLSLNSQLT*QVAGARSHDAGIYRHAT**R**EDVLGTSDALESEMLGHPKRECRFITCVFWDRID**RR**NSGVT

EDVISEDYEGPQGSVLRNLQEQ**KR**AINTL**RK**LTEILKPDQQQIVTT**RKR**TCRLRLGGHCLTEELDKAA**K**QYAYLKSG**K**SPG**R**

RRRETGNAAILENHDRYE

Supplementary Fig. 2. Post-translational processing of novel AG Peptide D prohormone observed by MALDI MS in individual dense core vesicles. SignalP[3] tool predicts signal sequence cleavage between residues 27 and 28: VAG-AR. However, our LC-MS/MS analysis identified an amidated peptide Q[24]-A[33] indicating that signal cleavage is most likely between 23 and 24: QLT-QV. We find that prohormone is cleaved into two major chains each featuring a disulfide bridge and conventional cleavage sites that result in production of various forms of cyclic peptides (Supplementary Table 1). The following mature peptides with PTMs were detected in individual DCVs by MALDI MS: E[42]-D[75], T[132]-D[147], T[132]-A[150] with disulfide bridge, T[132]-S[158], K[130]-P[162] and T[132]-P[162] with disulfide bridge and amidation, a C-terminus peptide Q [152]-P[162] with amidation, and linker peptides A[107]-T[128] and L[114-T[128] with no modifications. In addition, several truncated forms of mature peptides and a linear form of peptide T[132]-P[162] were detected. In total, 8 peptides from this new prohormone were identified and validated by LC-MS/MS.
Solid lines denote detected full-length mature peptides, confirmed cleavage sites are indicated in bold, PTM sites are colored: blue - amidation of preceding residue, green – disulfide bridges. Signal peptide sequence *italicized.*

Supplementary Table 1. AG Peptide D prohormone-supporting peptides detected by LC-MS/MS. Confidence in peptide identification represented by **-10lgP -** negative logarithm of probability; molecular mass error for measured versus predicted molecular mass of analyte in **ppm -** parts per million; PTM noted as follows: C-C disulfide bond; A – amidation; Ac – acetylation.

Accession	Protein name	Peptide	-10LgP	Mass	ppm	*m/z*	z	RT	Start	End	PTM
XP_012943319.1	Atrial gland D peptide	R.EDVLGTSDALESEMLGHPKREC(-1.01)RFITC(-1.01)VFWDRID.R	95.2	3964.85	1.7	793.9796	5	51.5407	42	75	C-C
XP_012943319.1	Atrial gland D peptide	E.DVLGTSDALESEMLGHPK.R	93.02	1897.91	1.9	949.9636	2	24.2915	43	60	
XP_012943319.1	Atrial gland D peptide	R.KRTC(-1.01)RLRLGGHC(-1.01)LTEELDKAAKQYAYLKSGKSP(-0.98).G	75.87	3716.97	1.2	744.4025	5	24.5948	130	162	A, C-C
XP_012943319.1	Atrial gland D peptide	R.KRTC(-1.01)RLRLGGHC(-1.01)LTEELDKAAKQYAYLKSGKSP.G	82.52	3717.96	13.3	744.6083	5	23.9245	130	162	C-C
XP_012943319.1	Atrial gland D peptide	R.TC(-1.01)RLRLGGHC(-1.01)LTEELDKAA.K	58.39	2083.03	3.6	695.3532	3	22.0478	132	150	C-C
XP_012943319.1	Atrial gland D peptide	R.TC(-1.01)RLRLGGHC(-1.01)LTEELDKAAKQYAYLKS(-0.98).G	87.49	3063.57	8.6	766.9075	4	29.7955	132	158	A, C-C
XP_012943319.1	Atrial gland D peptide	R.TC(-1.01)RLRLGGHC(-1.01)LTEELDKAAKQYAYLKSGKSP(-0.98).G	92.84	3432.78	7.9	687.5679	5	27.2446	132	162	A, C-C
XP_012943319.1	Atrial gland D peptide	K.QYAYLKSGKSP(-0.98).G	69.31	1239.66	4.4	1240.6738	1	23.2578	152	162	A

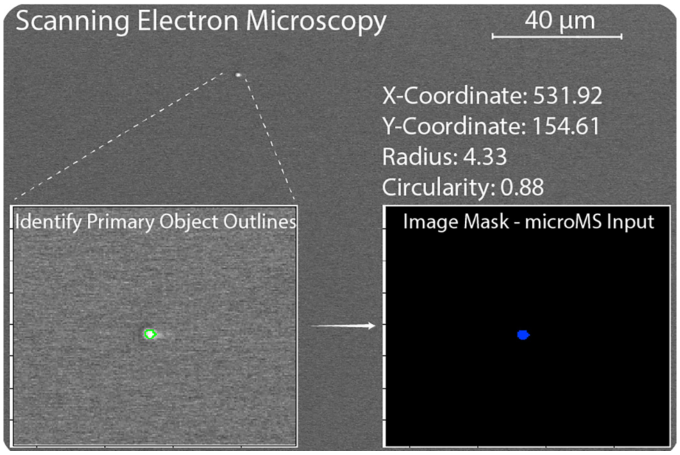

Supplementary Fig. 3. DCV recognition in scanning electron microscopy images. Application of pipeline for DCV recognition in images obtained with FEI Quanta FEG 450 ESEM. Generated by modified microMS approach, information on the x/y coordinates, radius and circularity displayed. Images were acquired using an accelerating voltage of 10 kV, dwell time of 10 µs, and working distance of 6.6 mm. A total of 98 ESEM images from one technical replicate were stitched together to form a composite image (not shown). From the composite image a total of 586 objects (DCVs) were identified.

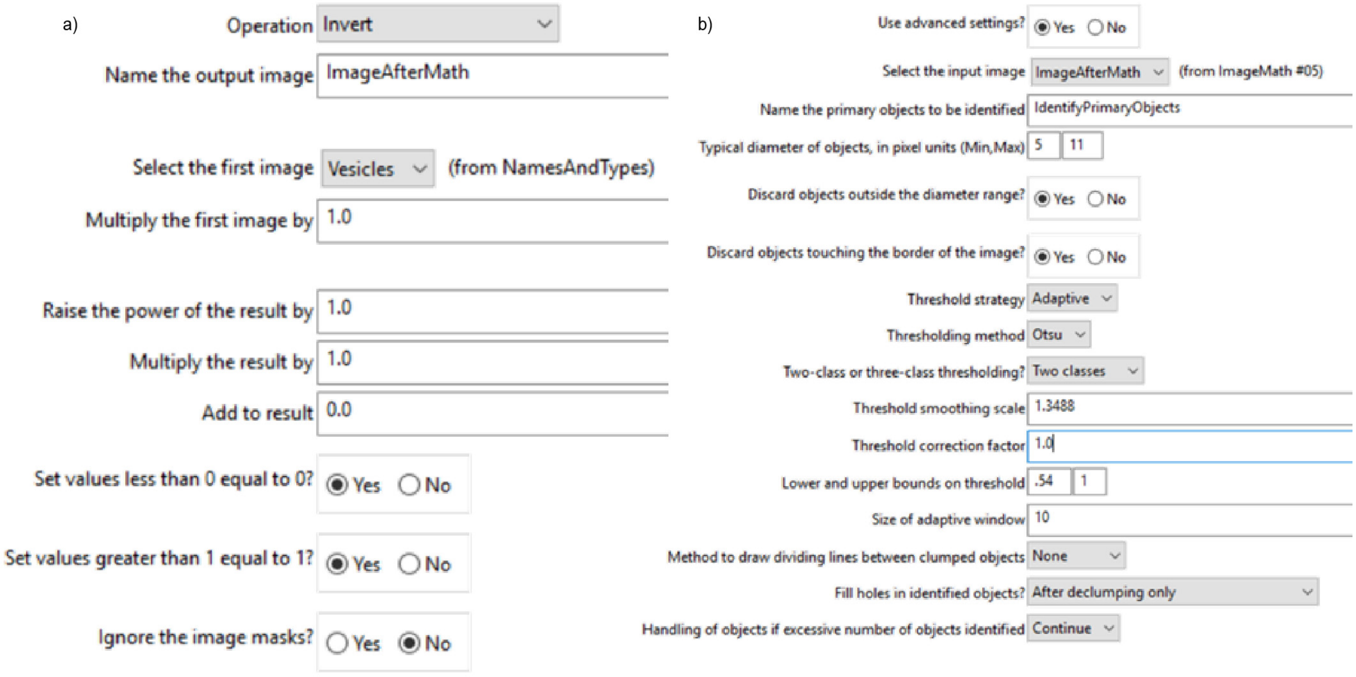

Supplementary Fig. 4. CellProfiler[4] parameters chosen for experiments. (a) ImageMath and (b) IdentifyPrimaryObject parameters used in the CellProfiler pipeline.

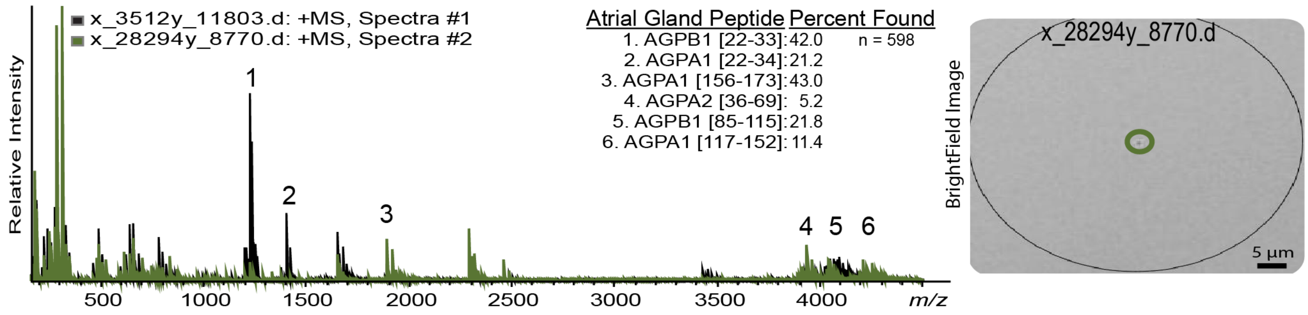

Supplementary Fig. 5. Representative mass spectra and brightfield DCV image. Two DCV mass spectra are overlaid, demonstrating the coverage of AG peptides, with a list detailing the detected AG peptides and their respective percent found out of the 598 DCVs analyzed. Each slide held DCVs from three animals (biological replicates) and a total of three slides (technical replicates) were prepared. Brightfield image with the DCV (x_28294y_8770.d) circled in green along with the corresponding mass spectrum, x_28294y_8770.d. The black circle represents the 100 µm MALDI laser spot size, allowing visual verification of single-vesicle targeting. Black scale bar = 5 µm.

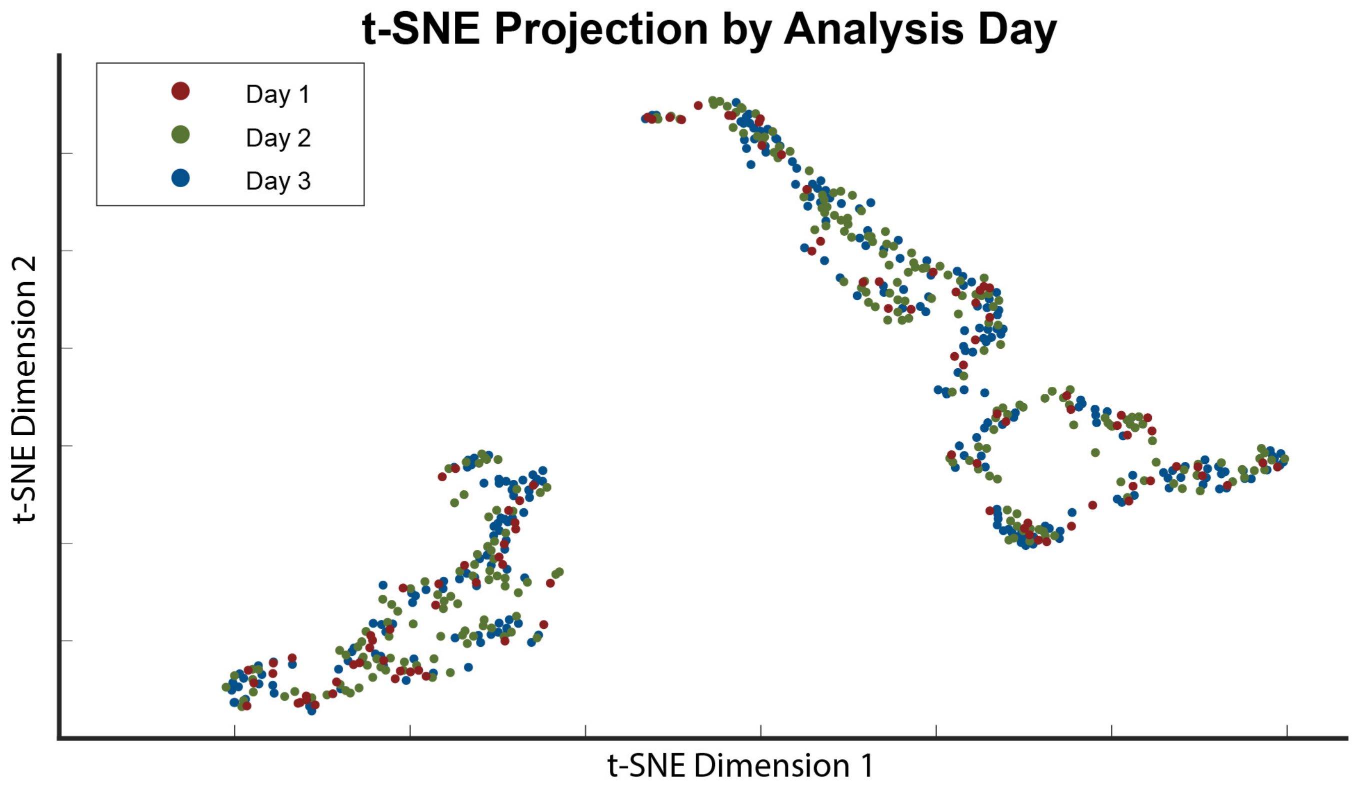

Supplementary Fig. 6. t-SNE projection by analysis day. t-SNE was used to visualize the DCV dataset colored by the analysis days to demonstrate that the clustering was not caused by non-biological variation, e.g., instrumental noise or biological factors such as individual animal variability. Both biological and non-biological variations often recognized as 'batch effects' can cause false clustering.

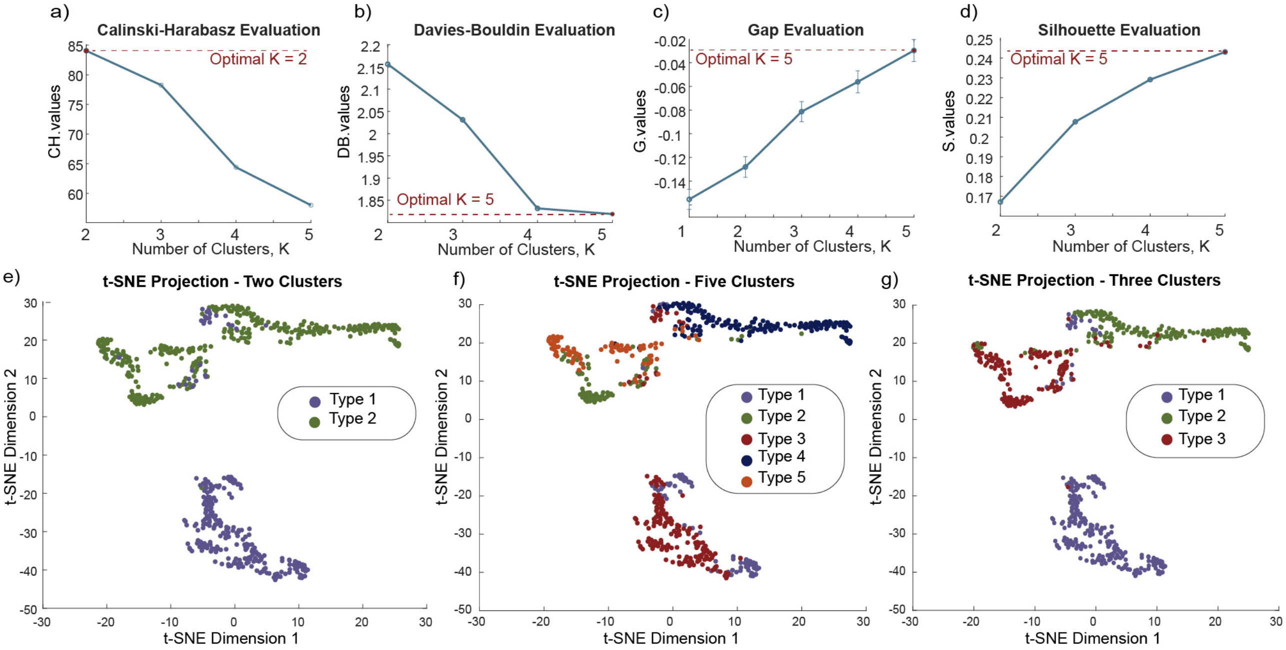

Supplementary Fig. 7. Clustering without data dimensionality reduction. Clustering analysis was performed using the entire dataset without performing molecular feature selection using CX decomposition. (a–d) Calinksi-Harabasz, Davies-Bouldin, Gap and Silhouette clustering were performed on the original dataset indicating the optimal k. The gap curve presented in (c) is displayed with ±1 standard error bars. (e–g) K-means was applied using the cosine similarity as a distance metric to generate DCV groups based off similar mass spectral features. t-SNE was then used to visualize the K-means cluster assigned index. Although, k = 2 or 5 were demonstrated to be the optimal cluster numbers, the t-SNE projection may support the possible appearance of a third cluster. A total of 598 DCVs were measured, where each slide held DCVs from three animals (biological replicates) and a total of three slides (technical replicates) were prepared.

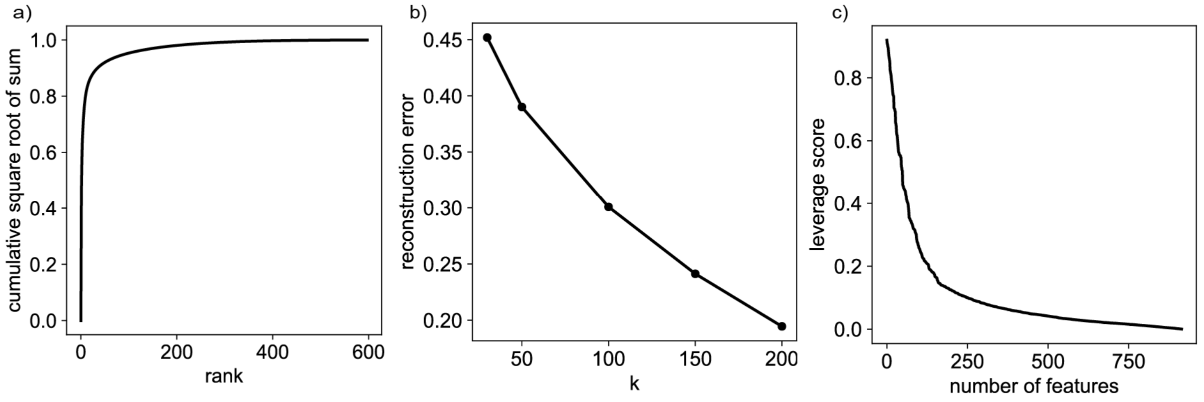

Supplementary Fig. 8. Singular value decomposition of the processed dataset prior to feature selection. (a) The cumulative square root of the sum of the obtained singular values. (b) The reconstruction error after rank truncation of the dataset using different k. (c) The distribution of the sorted leverage scores. These results help support the idea that the original high-dimensional dataset can be accurately approximated through a low-rank representation.

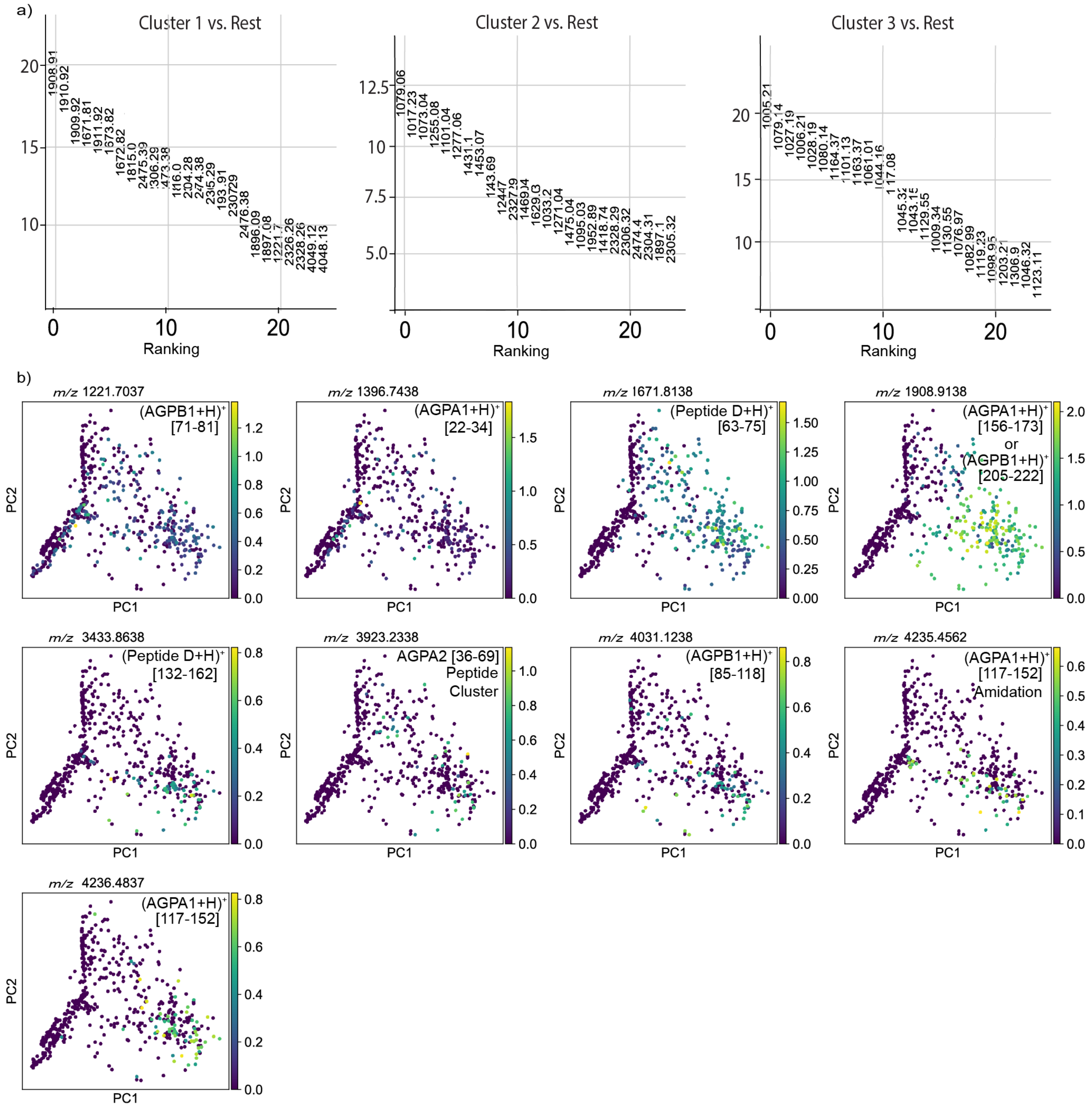

Supplementary Fig. 9. Wilcoxon rank-sum test and feature value distributions. Further analysis was performed on the dataset with the features selected via leverage scores. (a) The top 30 significantly different mass spectral features for each individual cluster were compared against the rest of the clusters using a two-sided Wilcoxon rank-sum test. The feature value distributions (log intensities) are shown in (b) for a subset of features, which are distinctly localized according to their cluster assignments. Known AG peptides are labeled where the localization of the $[M+H]^+$ features follow their corresponding adducts, which are shown in the main text. Features with higher intensity values are often ranked to be of higher importance than features of lower intensity and this is demonstrated in the main text by the propensity of the model to choose features belonging to the more intense sodiated adducts versus the less intense $[M+H]^+$ ions.

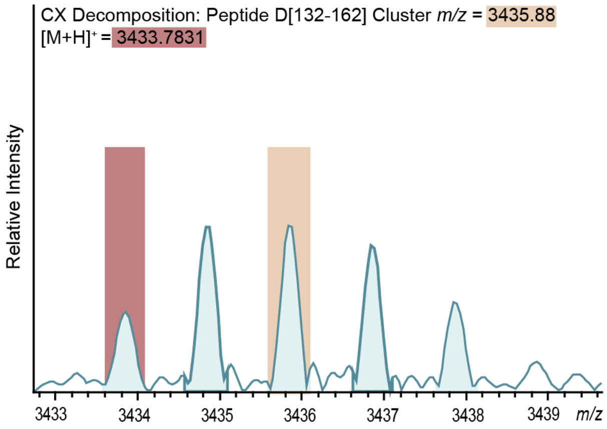

Supplementary Fig. 10. CX decomposition-identified peptide cluster. The monoisotopic ion is not always the most abundant isotope type in a mass spectrum, which may influence the propensity of the CX decomposition model to select a feature with a higher intensity value versus a feature with a lower intensity value and this is shown with the Peptide D[132-162] peptide cluster where the CX decomposition-selected peak is highlighted in tan and the less intense $[M+H]^+$ ion is highlighted in red. All peptides were confirmed with LC-MS/MS using peptide extracts from AG homogenates (n = 3).

Supplementary Table 2. LC-MS/MS validation of peptides detected in AG DCVs by MALDI MS. The mass error in ppm for MALDI MS measurements as well as the frequency each peptide was detected out of the population of 598 individual DCVs is given as a percentage. Only peptides measured by MALDI MS within our mass accuracy tolerance of ± 5 ppm and that were also identified in the AG homogenates by LC-MS/MS are included in the table. The confidence in peptide identification by LC-MS/MS is represented by -10lgP - negative logarithm of probability; molecular mass error for measured versus predicted molecular mass of analyte in ppm - parts per million; MALDI mass match error ≤ 5ppm; PTM noted as follows: pGlu (Q) – pyroglutamylation from glutamine; pGlu (E) – pyroglutamylation from glutamic acid; C-C disulfide bond; A – amidation; Ac – acetylation, P – phosphorylation, O – oxidation. %DCV indicates percentage of vesicles where identified peptides were detected; 0.17% of DCVs corresponds to detection in a single DCV and assignment by MALDI MS remains putative.

Accession	Protein name	Peptide	Unique	-10lgP	Mass	LC-MS/MS [ppm]	*m/z*	z	RT	PTM	MALDI MS [ppm]	%DCV
NP_001191518.1	Feeding circuit activating peptides precursor	R.FDNSAGE.K	Y	52.91	738.28	-1.1	739.29	1	8.03		-4.41	14.21
NP_001191518.1	Feeding circuit activating peptides precursor	R.QVDRLGGFQVHGW.K	Y	67.27	1497.75	-4.9	749.88	2	20.67		-2.85	1.17
NP_001191561.1	Cerebral peptide 1 precursor	E.STDNVVLSSSPDSQKAATS.R	Y	56.14	1892.90	-6.4	947.45	2	10.32		2.39	6.52
NP_001191561.1	Cerebral peptide 1 precursor	S.SESTDNVVLSSSPDSQKAATS.R	Y	66.95	2108.97	-4.8	1055.49	2	10.44		0.01	0.33
NP_001191670.1	ELH precursor	R.ISINQDLKAITDMLLTEQI(-0.98).R	N	43.82	2157.17	0	1079.60	2	67.59	A	-4.85	4.35
NP_001191670.1	ELH precursor	R.ISINQDLKAITDMLLTEQIRERQRYLADLRQRLLE.K	Y	60.08	4254.33	0.4	710.06	6	55.35		-0.28	2.34
NP_001191670.1	ELH precursor	R.ISINQDLKAITDMLLTEQIRERQRYLADLRQRLLEK(-0.98).G	Y	69.35	4381.44	9.7	877.31	5	49.39	A	-4.47	0.17
NP_001191670.1	ELH precursor	R.SVLTPSLSSLGESLESGIS.K	N	94.14	1861.95	8.5	931.99	2	46.41		3.45	0.67
XP_005098921.1	Uncharacterized protein LOC101856965	R.PFDKISSSGFRGF(-0.98).G	Y	44.66	1442.73	1.6	481.92	3	18.24	A	-3.24	3.68
XP_005099655.1	Uncharacterized protein LOC101850568	R.NRLEILDMLKELDRQAKVAA.K	Y	47.5	2325.28	2.2	582.33	4	36.74		-4.42	35.62
XP_005099655.1	Uncharacterized protein LOC101850569	R.WSDPISQNEEGANSISLRNRLEILDMLKELDRQA.K	Y	73.78	3939.97	-4.9	789.00	5	51.12		3.43	2.01
XP_012943319.1	Atrial gland peptide D	E.DVLGTSDALESEMLGHPK.R	Y	101.61	1897.91	-1.7	949.96	2	24.29		4.91	0.5
XP_012943319.1	Atrial gland peptide D	K.Q(-17.03)YAYLKSGKSP(-0.98).G	Y	60.1	1222.63	-2.4	612.33	2	11.46	A, pGlu (Q)	1.08	0.5
XP_012943319.1	Atrial gland peptide D	L.DKAAKQYAYLKSGKSP(-0.98).G	Y	90.84	1752.95	-3.2	877.48	2	23.12	A	-5.94	16.89
XP_012943319.1	Atrial gland peptide D	R.AINTLRKLTEILKPDQQQIVTT.R	Y	68.53	2522.44	0.4	841.83	3	29.49		2.11	2.17
XP_012943319.1	Atrial gland peptide D	R.EDVLGTSDALESEMLGHPKREC(-1.01)RFITC(-1.01)VFWDRID.R	Y	67.13	3964.85	-2.9	793.98	5	52.31	C-C		
XP_012943319.1	Atrial gland peptide D	R.TC(-1.01)RLRLGGHC(-1.01)LTEELD.K	Y	38.21	1812.86	4.1	605.30	3	23.33	C-C	-1.47	0.17
XP_012945134.1	Atrial gland and califin peptides-like	K.E(-18.01)DGAQPYFMTPRLRFYPI(-0.98).G	N	40.59	2181.08	-2.5	1091.55	2	29.21	A, pGlu (E)	-2.1	2.17
XP_012945134.1	Atrial gland and califin peptides-like	K.EDGAQPYFMTPRLRFYPI(-0.98).G	N	74.34	2199.09	3.7	1100.56	2	43.53	A	-3.44	4.35
XP_012945134.1	Atrial gland and califin peptides-like	R.A(+42.01)VKLSSDGNYPFDLSKEDGAQPYFMTPRLRFYPI(-0.98).G	Y	51.15	3962.97	8.4	991.76	4	49.64	A, Ac	2.98	2.17
XP_012945134.1	Atrial gland and califin peptides-like	R.AAGEMEQSEGQNPETKSHSW.R	N	90.18	2201.93	-1.3	734.99	3	9.94		1.1	0.84
XP_012945134.1	Atrial gland and califin peptides-like	R.AVKLSSDGNYPFDLS(+79.97)KEDGAQPYFMTPRLRFYPI(-0.98).G	Y	37.41	4000.92	10.4	1001.25	4	45.35	A, P	4.63	0.67

XP_012945134.1	Atrial gland and califin peptides-like	R.AVKLSSDGNYPFDLSK(+42.01)EDGAQPYFMTPRLRFYPIGK.R	Y	41.09	4149.07	7.9	1038.29	4	47.74	Ac	-4.45	0.17
XP_012945134.1	Atrial gland and califin peptides-like	R.AVKLSSDGNYPFDLSKEDGAQPYFM(+15.99)TPRLRFYPI(-0.98).G	Y	63.73	3936.95	-0.9	788.40	5	43.91	A, O	0.01	1.67
XP_012945134.1	Atrial gland and califin peptides-like	R.AVKLSSDGNYPFDLSKEDGAQPYFMTPRLRFYPI(-0.98).G	Y	98.97	3920.96	0.3	785.20	5	39.50	A	0.03	0.33
XP_012945134.1	Atrial gland and califin peptides-like	R.AVKLSSDGNYPFDLSKEDGAQPYFMTPRLRFYPIG.K	Y	85.52	3978.96	-2.4	796.80	5	43.46		-3.26	1.17
XP_012945134.1	Atrial gland and califin peptides-like	R.DSDVSLFNGDLLPNGRCS	N	84.74	1907.87	0.4	954.95	2	54.66		2.68	42.47
XP_012945134.1	Atrial gland and califin peptides-like	R.DSDVSLFNGDLLPNGRCS(-0.98)	N	53.48	1906.88	-3.9	954.45	2	29.80	A	-4.39	23.24
XP_012945134.1	Atrial gland and califin peptides-like	R.ISINQDLK(+42.01)AITDMLLTEQIQARRRCLAALRQRLLDL(-0.98).G	N	46.53	4232.37	9.7	1059.11	4	67.60	A, Ac	-2.17	0.5
XP_012945134.1	Atrial gland and califin peptides-like	R.SVLTPSLSSLGESLESGIS.K	N	94.14	1861.95	8.5	931.99	2	46.41		3.45	0.67
XP_012945142.1	Atrial gland peptide B	K.EDGAQPYFMTPRLRFYPI(-0.98).G	N	74.34	2199.09	3.7	1100.56	2	43.53	A	-3.44	4.35
XP_012945142.1	Atrial gland peptide B	R.A(+42.01)VKSSSYEKYPFDLSKEDGAQPYFMTPRLRFYPI(-0.98).G	N	71.45	4071.02	1.6	815.22	5	32.26	A, Ac	1.73	4.68
XP_012945142.1	Atrial gland peptide B	R.AAGGMEQSEGQNPETKSHSWRE.R	N	87.93	2415.05	-4.7	806.02	3	9.15		4.11	0.17
XP_012945142.1	Atrial gland peptide B	R.AVKSSSYEKYPFDLSKEDGAQPYFM(+15.99)TPRLRFYPI(-0.98).G	N	82.37	4045.01	0	810.01	5	25.63	A, O	-1.2	4.35
XP_012945142.1	Atrial gland peptide B	R.AVKSSSYEKYPFDLSKEDGAQPYFMTPRLRFYPI(-0.98).G	N	105.29	4029.01	4.6	806.82	5	34.67	A	1.25	2.01
XP_012945142.1	Atrial gland peptide B	R.AVKSSSYEKYPFDLSKEDGAQPYFMTPRLRFYPI.G	N	67.48	4030.00	14.6	807.02	5	30.25		-2.28	0.84
XP_012945142.1	Atrial gland peptide B	R.AVKSSSYEKYPFDLSKEDGAQPYFMTPRLRFYPIG.K	N	73.56	4087.02	-6.9	818.41	5	30.14		-1.93	2.34
XP_012945142.1	Atrial gland peptide B	R.DSDVSLFNGDLLPNGRCS	N	84.74	1907.87	0.4	954.95	2	54.66		2.68	42.47
XP_012945143.1	Atrial gland and califin peptides-like	K.E(-18.01)DGAQPYFMTPRLRFYPI(-0.98).G	N	40.59	2181.08	-2.5	1091.55	2	29.21	A, pGlu (E)	-2.1	2.17
XP_012945143.1	Atrial gland and califin peptides-like	K.EDGAQPYFMTPRLRFYPI(-0.98).G	N	74.34	2199.09	3.7	1100.56	2	43.53	A	-3.44	4.35
XP_012945143.1	Atrial gland and califin peptides-like	R.A(+42.01)VKSSSYENYPFDLSKEDGAQPYFMTPRLRFYPI(-0.98).G	Y	35.23	4056.97	8.6	812.41	5	32.24	A, Ac	-3.94	0.84
XP_012945143.1	Atrial gland and califin peptides-like	R.AAGEMEQSEGQNPETKSHSW.R	N	90.18	2201.93	-1.3	734.99	3	9.94		1.1	0.84
XP_012945143.1	Atrial gland and califin peptides-like	R.AVKSSSYENYPFDLS.K	Y	61.5	1705.78	-0.1	853.90	2	20.48		4.29	7.69
XP_012945143.1	Atrial gland and califin peptides-like	R.AVKSSSYENYPFDLSKED(-0.98).G	Y	35.08	2076.96	-9.7	693.32	3	16.53	A	4.58	0.17
XP_012945143.1	Atrial gland and califin peptides-like	R.AVKSSSYENYPFDLSKEDGAQPYFM(+15.99)TPRLRFYPI(-0.98).G	Y	51.54	4030.96	2	807.20	5	36.87	A, O	1.15	0.67
XP_012945143.1	Atrial gland and califin peptides-like	R.AVKSSSYENYPFDLSKEDGAQPYFMTPRLRFYPI(-0.98).G	Y	88.07	4014.96	3.1	1004.75	4	35.70	A	-0.12	1.84
XP_012945143.1	Atrial gland and califin peptides-like	R.DSDVSLFNGDLLPNGRCS	N	84.74	1907.87	0.4	954.95	2	54.66		2.68	42.47
XP_012945143.1	Atrial gland and califin peptides-like	R.ISINQDLK(+42.01)AITDMLLTEQIQARRRCLAALRQRLLDL(-0.98).G	N	46.53	4232.37	9.7	1059.11	4	67.60	A, Ac	-2.17	0.5
XP_012945143.1	Atrial gland and califin peptides-like	R.ISINQDLKAITDMLLTEQIQARRRCLAALRQRLLDL(-0.98).G	N	65.69	4190.36	9	699.41	6	57.44	A	2.77	2.84
XP_012945143.1	Atrial gland and califin peptides-like	R.SVLTPSLSSLGESLESGIS.K	N	94.14	1861.95	8.5	931.99	2	46.41		3.45	0.67

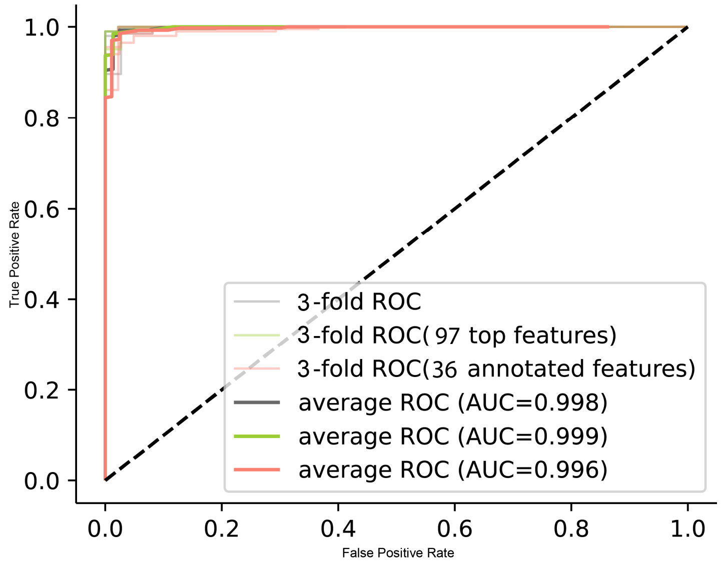

Supplementary Fig. 11. ROC curves obtained from 3-fold validation. The grey ROC curve represents the classification using all of the mass spectral features. Green ROC curve represents classification using the 97 top features. Orange ROC curves represent the classification using the 36 annotated features. The ROC curves demonstrate that vesicle classification can be performed with a much smaller subset of features identifed by SHAP values.

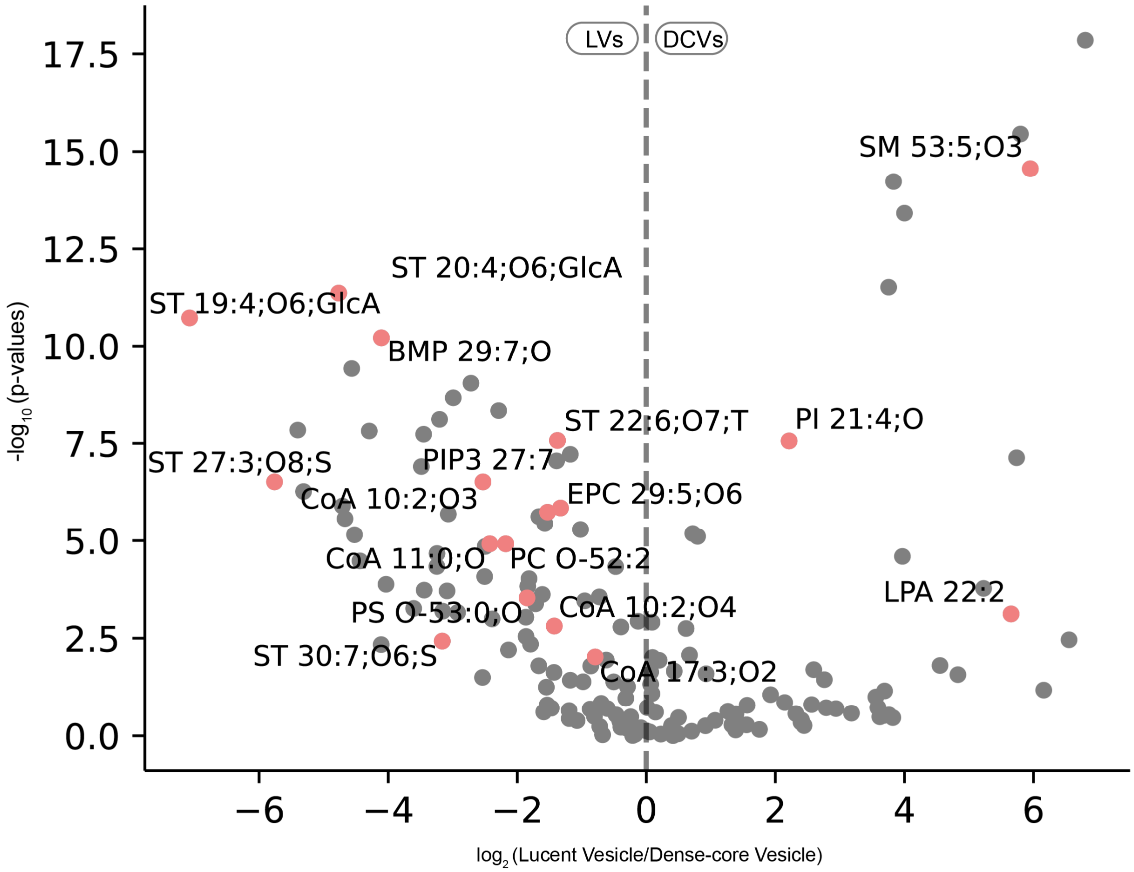

Supplementary Fig. 12. Volcano plot of the differential lipid features between DCVs and LVs. The x-axis shows the $\log_2$ ratios of the average spectral signal intensity between DCVs and LVs, versus the $-\log_{10}$ p-values of the spectral profiles on the y-axis. The pink colored dots represent the putatively identified lipids using LIPID MAPS[5] database. Data points with high significance (p-value) appear higher on the y-axis. The x-axis represents the fold change between LVs and DCVs. Therefore, highly significant features with large fold changes in the LVs will appear on left-hand, topside of the plot. Data for 598 DCVs and 123 LVs are presented. A two-sided Wilcoxon rank-sum test was used and the unadjusted p-values provided.

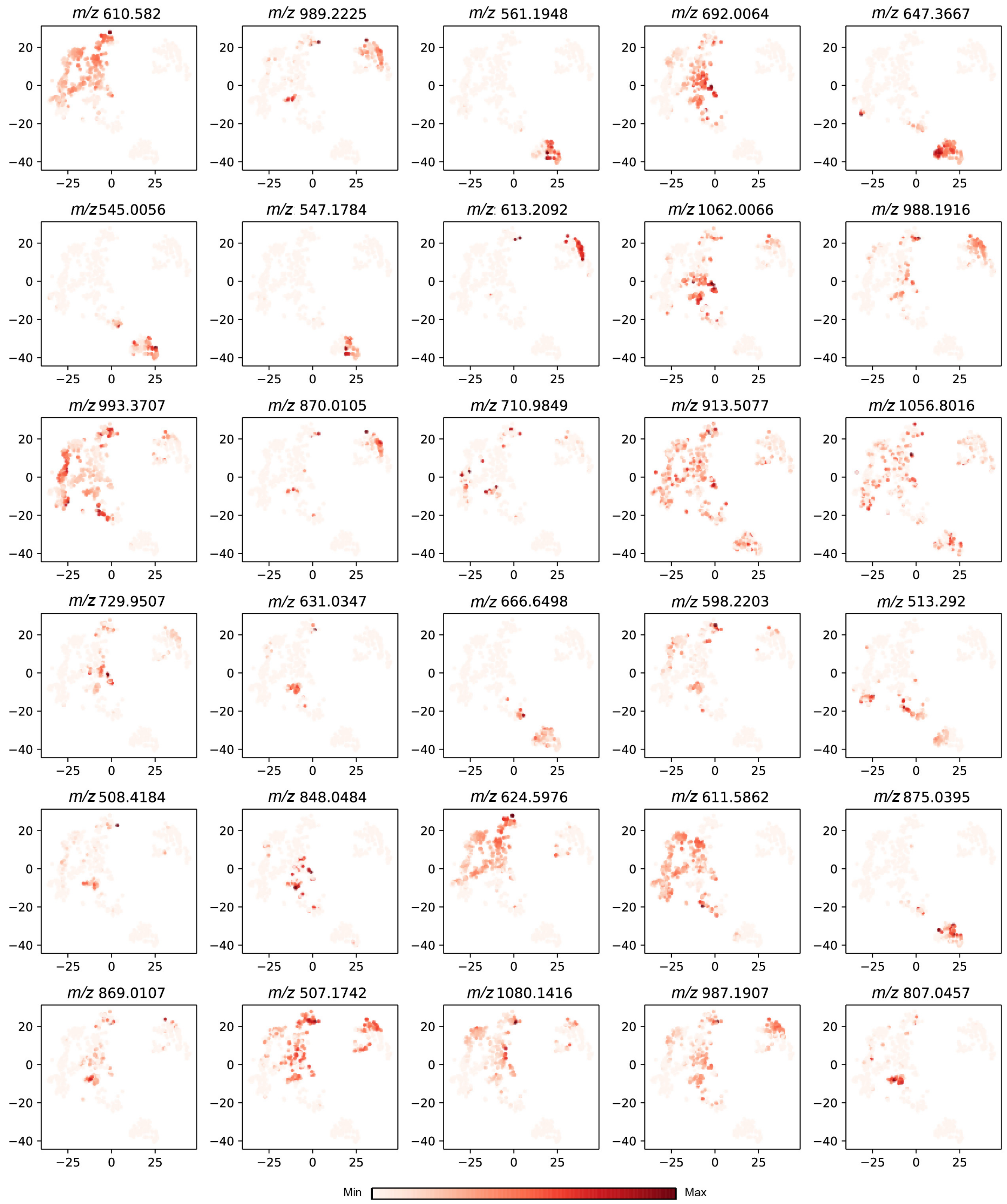

Supplementary Fig. 13. DCV and LV t-SNE feature distributions. Differential feature distributions were overlaid onto the t-SNE plot. Distributions for the top contributing features for the vesicle classification task that were identified by SHAP are shown in descending importance from top-to-bottom and left-to-right. Colors indicate the normalized intensities of corresponding *m/z* peaks.

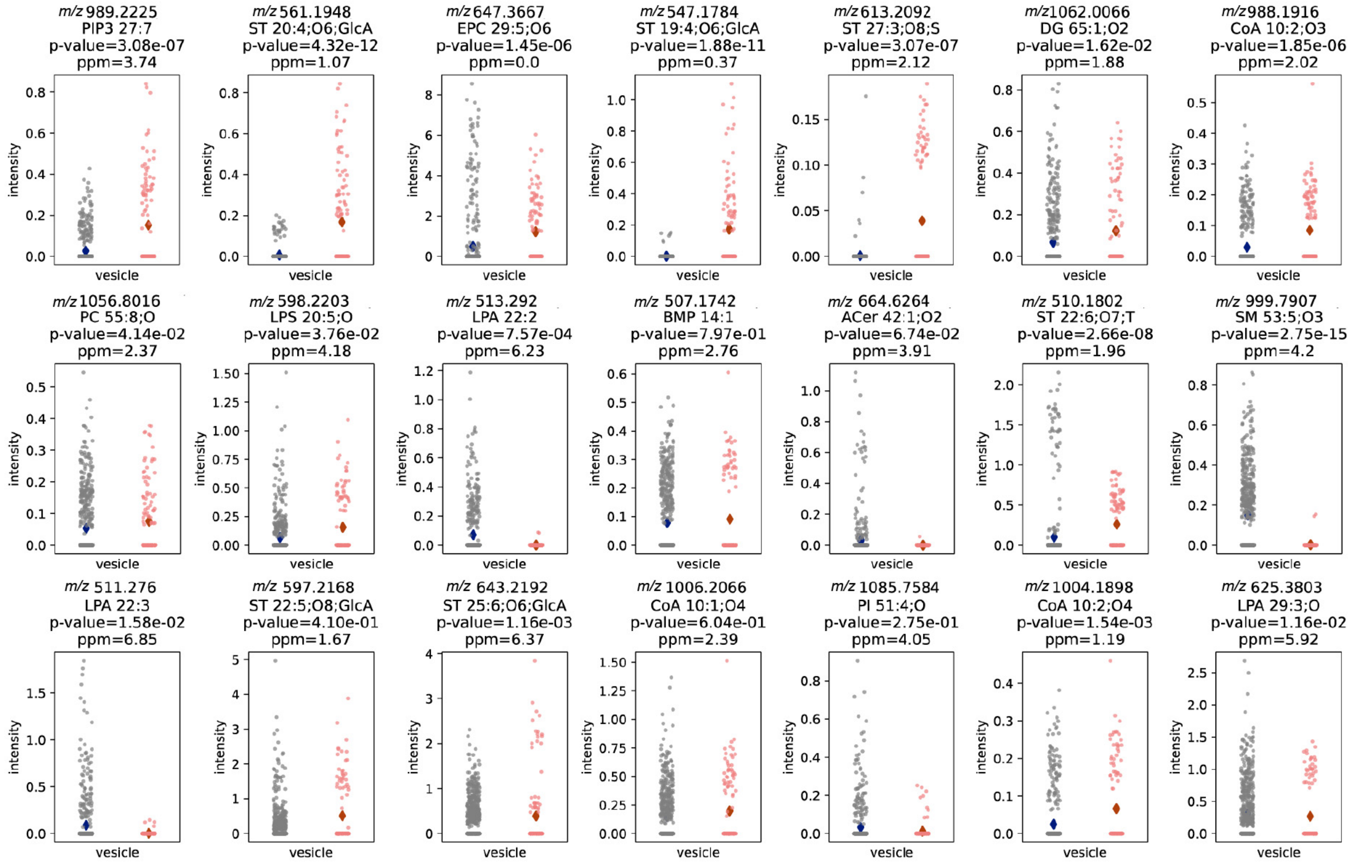

Supplementary Fig. 14. Mass spectral profile comparisons between DCVs (gray) and LVs (pink). The top contributing features that were identified by SHAP and their identities putatively assigned by searching against LIPID MAPS[5] with a 7 ppm tolerance are shown. The comparisons support the enrichment in sterol lipids in the LVs when compared to the DCVs. Relative signal intensities are plotted on the y-axis. Each point corresponds to measurement from a single vesicle. Data for 598 DCVs and 123 LVs are presented. A two-sided Wilcoxon rank-sum test was used and the unadjusted p-values provided.

References

1. Sievers F, Wilm A, Dineen DG, Gibson TJ, Karplus K, Li W, Lopez R, McWilliam H, Remmert M, Söding J, Thompson JD, Higgins DG. Fast, scalable generation of high-quality protein multiple sequence alignments using Clustal Omega. *Molecular Systems Biology* 7 (2011):539.

2. Nagle, G. T., S. D. Painter, J. E. Blankenship, and A. Kurosky. Proteolytic Processing of Egg-Laying Hormone-Related Precursors in Aplysia. Identification of Peptide Regions Critical for Biological Activity. *Journal of Biological Chemistry* 263 (1988): 9223–37.

3. Almagro Armenteros, José Juan, Konstantinos D. Tsirigos, Casper Kaae Sønderby, Thomas Nordahl Petersen, Ole Winther, Søren Brunak, Gunnar von Heijne, and Henrik Nielsen. SignalP 5.0 Improves Signal Peptide Predictions Using Deep Neural Networks. *Nature Biotechnology* 37 (2019): 420–23.

4. Jones TR, Kang IH, Wheeler DB, Lindquist RA, Papallo A, Sabatini DM, Golland P, Carpenter AE CellProfiler Analyst: data exploration and analysis software for complex image-based screens. *BMC Bioinformatics 9* (2008):482.

5. Fahy E, Subramaniam S, Murphy R, Nishijima M, Raetz C, Shimizu T, Spener F, van Meer G, Wakelam M and Dennis E.A. Update of the LIPID MAPS® comprehensive classification system for lipids. *Journal of Lipid Research* 50 (2009): S9–S14.
