# Supplementary material for: Image-guided MALDI mass spectrometry for high-throughput single-organelle characterization
Source: Nat Methods. 2021 Sep 30;18(10):1233–8. doi: 10.1038/s41592-021-01277-2 (PMC8490150; doi:10.1038/s41592-021-01277-2)
Supplement: Supplementary file 2 — Reporting Summary [file 41592_2021_1277_MOESM2_ESM.pdf]

nature research

Corresponding author(s): Jonathan Sweedler

Last updated by author(s): Jul 14, 2021

Reporting Summary

Nature Research wishes to improve the reproducibility of the work that we publish. This form provides structure for consistency and transparency in reporting. For further information on Nature Research policies, see our Editorial Policies and the Editorial Policy Checklist.

Statistics

For all statistical analyses, confirm that the following items are present in the figure legend, table legend, main text, or Methods section.

n/a	Confirmed	
☐	☒	The exact sample size (*n*) for each experimental group/condition, given as a discrete number and unit of measurement
☐	☒	A statement on whether measurements were taken from distinct samples or whether the same sample was measured repeatedly
☐	☒	The statistical test(s) used AND whether they are one- or two-sided<br>*Only common tests should be described solely by name; describe more complex techniques in the Methods section.*
☐	☒	A description of all covariates tested
☐	☒	A description of any assumptions or corrections, such as tests of normality and adjustment for multiple comparisons
☐	☒	A full description of the statistical parameters including central tendency (e.g. means) or other basic estimates (e.g. regression coefficient) AND variation (e.g. standard deviation) or associated estimates of uncertainty (e.g. confidence intervals)
☒	☐	For null hypothesis testing, the test statistic (e.g. *F*, *t*, *r*) with confidence intervals, effect sizes, degrees of freedom and *P* value noted<br>*Give P values as exact values whenever suitable.*
☒	☐	For Bayesian analysis, information on the choice of priors and Markov chain Monte Carlo settings
☒	☐	For hierarchical and complex designs, identification of the appropriate level for tests and full reporting of outcomes
☒	☐	Estimates of effect sizes (e.g. Cohen's *d*, Pearson's *r*), indicating how they were calculated

Our web collection on statistics for biologists contains articles on many of the points above.

Software and code

Policy information about availability of computer code

Data collection	Optical microscopy images were acquired using ZEN 2.0 Pro edition (Zeiss, Jena, Germany) software. Scanning electron microscopy images were acquired using xT Microscope Control 6.2.8.3161 (FEI, Hillsboro, Oregon). The microscopy images were processed using CellProfiler 3.1.8 (Broad Institute, Cambridge, Massachusetts), ImageJ 1.53c (NIH & Laboratory for Optical and Computational Instrumentation, University of Wisconsin) and MicroMS (University of Illinois at Urbana-Champaign, Urbana, Illinois). The MALDI mass spectrometry data was acquired using ftmsControl 2.2.0 (Bruker Corporation, Billerica, Massachusetts) . The LC-MS/MS data was acquired using oTOFControl 6.2 (Bruker Corporation, Billerica, Massachusetts) and Hystar 5.1 (Bruker Corporation, Billerica, Massachusetts).
Data analysis	Raw MALDI MS data were preprocessed using DataAnalysis 4.4.2 (Bruker Corporation, Billerica, Massachusetts) and in-house developed MATLAB scripts R2018b (MathWorks, Inc., Natick, Massachusetts). The data analysis pipeline was implemented in Python 3.7.1 (Python Software Foundation, Beaverton, Oregon) with open-source packages NumPy 1.19.2 (NumFOCUS, Inc., Austin, Texas), SciPy 1.5.3 (SciPy developers), scikit-learn 0.23.1 (scikit-learn developers), and single cell analysis package Scanpy 1.5.1. Plotting packages include matplotlib 3.3.2 (NumFOCUS, Inc., Austin, Texas) and seaborn 0.11.0. LC-MS/MS raw files were preprocessed using DataAnalysis 5.3 (Bruker Corporation, Billerica, Massachusetts) and PEAKS Online x1.3 (Bioinformatics Solutions Inc., Waterloo, Ontario). SignalP 5.0 (Bioinformatic Unit, Technical University of Denmark, Denmark) was used to predict sequence cleavages. Clustal Omega 1.2.4 (University College Dublin, Dublin, Ireland) was used for sequence alignment.

For manuscripts utilizing custom algorithms or software that are central to the research but not yet described in published literature, software must be made available to editors and reviewers. We strongly encourage code deposition in a community repository (e.g. GitHub). See the Nature Research guidelines for submitting code & software for further information.

Data

Policy information about availability of data

All manuscripts must include a data availability statement. This statement should provide the following information, where applicable:

- Accession codes, unique identifiers, or web links for publicly available datasets
- A list of figures that have associated raw data
- A description of any restrictions on data availability

The data that supports the findings of this study are publicly available via the Illinois Data Bank: https://doi.org/10.13012/B2IDB-5949772_V1

Field-specific reporting

Please select the one below that is the best fit for your research. If you are not sure, read the appropriate sections before making your selection.

☒ Life sciences ☐ Behavioural & social sciences ☐ Ecological, evolutionary & environmental sciences

For a reference copy of the document with all sections, see nature.com/documents/nr-reporting-summary-flat.pdf

Life sciences study design

All studies must disclose on these points even when the disclosure is negative.

Sample size	To account for biological variability between animals we chose to use three biological replicates in our study. To minimize MALDI MS shot-to-shot variability and changes in instrumental conditions between analysis days, we placed dense-core vesicles from each of the three biological replicates on the same microscopy slide. A total of three microscopy slides were prepared using the three biological replicates and analyzed on separate days. By measuring 598 dense-core vesicles across the three microscopy slides, we believe we have mitigated any unwanted variation that may be attributed to biological variability between animals or variation attributed to changes in MALDI MS instrumentation conditions.
Data exclusions	No data was excluded from the study.
Replication	We performed the dense-core vesicle measurements using two types of mass analyzers (Fourier-transform ion cyclotron resonance mass spectrometer and time-of-flight mass spectrometer) where expected atrial gland peptides were detected using both types of mass analyzers. Every detected peptide by MALDI MS was structurally characterized using LC-MS/MS. Each experimental setup contained at least three biological replicates.
Randomization	Animals/Dense-core vesicles were not subjected to different treatments or interventions that would create the possibility of selection bias. Therefore, we believe randomization does not apply to this study.
Blinding	Group allocation due to different treatments or interventions did not occur during this study, which is why we believe blinding is not applicable to this study.

Reporting for specific materials, systems and methods

We require information from authors about some types of materials, experimental systems and methods used in many studies. Here, indicate whether each material, system or method listed is relevant to your study. If you are not sure if a list item applies to your research, read the appropriate section before selecting a response.

Materials & experimental systems

n/a	Involved in the study	
☒	☐	Antibodies
☒	☐	Eukaryotic cell lines
☒	☐	Palaeontology and archaeology
☐	☒	Animals and other organisms
☒	☐	Human research participants
☒	☐	Clinical data
☒	☐	Dual use research of concern

Methods

n/a	Involved in the study	
☒	☐	ChIP-seq
☒	☐	Flow cytometry
☒	☐	MRI-based neuroimaging

Animals and other organisms

Policy information about studies involving animals; ARRIVE guidelines recommended for reporting animal research

Laboratory animals	Aplysia californica

Wild animals	This study did not involve wild animals.
Field-collected samples	This study did not involve field-collected samples (the animals were from the Aplysia Research Facility, University of Miami).
Ethics oversight	Exempt from oversight because the animals used are mollusks / invertebrates. However, we followed published recommendations for the humane treatment of gastropod mollusks in research, including use of anesthesia.

Note that full information on the approval of the study protocol must also be provided in the manuscript.
